# Dental and Maxillofacial Emergency Algorithms in Swiss Emergency Departments

**DOI:** 10.3390/jcm12082952

**Published:** 2023-04-19

**Authors:** Adelita Sommacal, Roland Bingisser, Andreas Filippi, Mascha Bethke, Florian M. Thieringer, Claude Jaquiéry, Britt-Isabelle Berg

**Affiliations:** 1 Department of Oral and Cranio-Maxillofacial Surgery, University Hospital Basel, 4031 Basel, Switzerland; 2 Faculty of Medicine, University of Basel, 4031 Basel, Switzerland; 3 Emergency Department, University Hospital Basel, 4031 Basel, Switzerland; 4 Clinic of Oral Surgery and Center of Dental Traumatology, University Center for Dental Medicine UZB, University of Basel, 4058 Basel, Switzerland; 5 Swiss MAM Research Group, Department of Biomedical Engineering, University of Basel, 4123 Allschwil, Switzerland

**Keywords:** algorithm, dental trauma, maxillofacial emergency, survey, emergency treatment

## Abstract

This study aimed to evaluate the availability and use of dental and maxillofacial emergency algorithms in Swiss hospitals. A survey was performed among physicians at Swiss emergency departments (ED) and participants of the “36th Annual Meeting of the Society for Oral and Cranio-Maxillofacial Surgery”. Eighty-nine EDs in Switzerland were questioned about the availability and use of electronic algorithms in their hospitals. Eighty-one (91%) participated in the study. In 75 (93%) of the EDs, electronic algorithms are used, mainly “medStandards”. Six have no available algorithms. Fifty-two (64%) use algorithms daily. Eight (10%) Swiss EDs have maxillofacial and dental algorithms, and 73 (90%) have no access to or do not know about them. For dental algorithms, 28 (38%) of the respondents would like to have access, and 16 (22%) do not desire access. For maxillofacial algorithms, 23 (32%) want to have access and 21 (29%) do not want it. Most (74%) of the participating maxillofacial surgeons did not know about the existence of ED algorithms regarding their specialty. Our study shows that the existence of specific algorithms is often not known. Furthermore, there is a demand for dental and maxillofacial algorithms in Swiss EDs.

## 1. Introduction

Fractures of the viscerocranium are the third most common fractures observed in Switzerland [1]. Facial trauma in general, with a prevalence of 10.4%, represents a substantial part of all traumas documented in Switzerland [1]. Globally, facial trauma is one of the most common injuries [2,3].

Odontogenic infections are also common reasons for emergency admissions [4]. Over 90% of all infections in the oral and maxillofacial region are of odontogenic origin [5]. Caries, dental trauma, pericoronitis, periodontal diseases, and insufficient endodontic treatment mainly lead to these infections [4,6]. The spreading of odontogenic abscesses can vary from localized submucosal abscesses up to life-threatening abscesses in the complete head and neck region. The most often affected spaces are the submandibular, the buccal space, and the canine fossa [7]. In patients with odontogenic abscesses, trismus can occur as a sign of involvement of the masticatory muscles. Several predisposing factors may influence the clinical course of odontogenic infections, such as immunodeficiency (e.g., due to human immunodeficiency virus (HIV) or chemotherapy) and diabetes mellitus. Physicians need to be aware of potentially life-threatening complications such as sepsis, airway obstruction, and cavernous sinus thrombosis. The therapeutic principle of suppurative infections, which implies that pus has to be drained, goes back to Hippocrates; this principle also applies to odontogenic infections and is still applicable today [8]. Algorithms can be beneficial in pointing out potential complications, especially to young physicians who are not trained in dental medicine and might not be familiar with the possible life-threatening conditions of odontogenic infections.

Furthermore, dental trauma has a considerable relevance in public health due to its high incidence [9] and its impact on the patients’ quality of life [10]. Petti et al. showed in their study that more than one billion living people have a history of dental trauma and concluded that dental trauma would rank in fifth place of the world’s most frequent acute and chronic diseases and injuries if listed [9]. Unfortunately, there is a lack of knowledge about the management of dental trauma among physicians in emergency departments (EDs) [11,12]. Patel et al. [11] stated in their article that there is a lack of dental education in medical studies. They suggested the use of algorithms for the management of common dental issues [11]. This may also apply to Switzerland and other countries.

Various sources can be used to look up medical know-how. Due to the existence of a broad spectrum of online sources such as digitalized textbooks, an enormous number of scientific articles, and videos on various platforms, information has become widely available, as have algorithms. Different types of medical algorithms exist for different purposes. There are clinical algorithms designed as diagnostic, therapeutic, or administrative workflows in the form of a flowchart or a decision tree. There are algorithms using statistical techniques, artificial intelligence, or machine learning technologies, such as in (personalized) risk assessment, for example [13,14].

Existing guidelines in the field of oral and maxillofacial surgery, as published by the “DGMKG” (German Society of cranio-maxillofacial surgery) on the platform of the “AWMF” (Association of the Scientific Medical Societies in Germany) [15], provide the current state of knowledge concerning treatment procedures. However, not all guidelines are currently up to date. They offer specific treatment information and seem to be more targeted towards specialized practitioners. General information on how to approach dental and maxillofacial emergencies is rarely stated; therefore, such guidelines may not be best suited for the needs of a general practitioner in the ED. In emergency situations, checklists can support less experienced practitioners in maintaining an overview and can reduce the risk of prejudicial conclusions being made due to a lack of assessment. These checklists can be created for medical purposes and can be integrated into algorithms.

**Figure 1:**
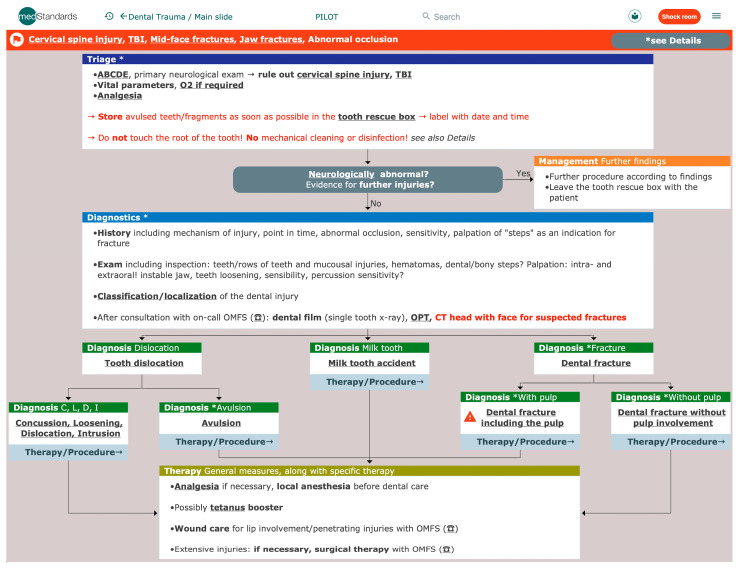
Screenshot ”medStandards” [16]—Dental Trauma. ABCDE: mnemonic for primary survey; TBI: traumatic brain injury; O_2_: oxygen; OPT: orthopantomogram; CT: computed tomography; OMFS: oral and maxillofacial surgeon.

Emergency algorithms, such as those offered by “medStandards” [16], can provide a quick overview of specific diagnoses. They are a helpful tool in EDs since they support decision making for an efficient and well-targeted triage, diagnostics, and therapy. “MedStandards” offers over 1000 pages of interactively linked evidence- and guideline-based algorithms [16]. In total, nine algorithms are currently available online in the field of facial trauma, odontogenic abscesses, dental and maxillofacial imaging, and dental trauma (Figure 1). Facial trauma is subdivided into six algorithms: blunt midface trauma, differential diagnosis in fractures of the viscerocranium, LeFort fractures, zygomatic and orbital fractures, mandibular fractures, and nasal bone fractures. The design and structure for all stated algorithms are the same, leading from the first contact to suggested diagnostics, diagnosis, therapies, and procedures. These algorithms are developed for the support of all disciplines.

Already in 1980, Looney et al. published a paper about algorithms in a research setting and suggested modes for adaptation to clinical settings [17]. Meanwhile, various algorithms are available for several fractures and emergencies from different providers [16,18,19,20].

To the best of our knowledge, there have been no data published about the use of dental and maxillofacial algorithms so far, despite maxillofacial and dental emergencies being daily business in larger units. Therefore, we aimed to evaluate the availability and use of dental and maxillofacial emergency algorithms in Swiss hospitals. With this gained knowledge, we hope to be able to provide improved support towards EDs.

## 2. Materials and Methods

A survey among doctors in Swiss EDs was performed primarily by telephone and alternatively by e-mail. All questions are presented in Table 1. The eligible hospitals were selected based on the published data from the Federal Office of Public Health (FOPH) for key figures on Swiss Hospitals in 2020 [21]. A total of 89 EDs were included, covering general hospitals from level 1 (university hospital) to level 5 (community hospital) with a surgical ED [22]. Specialized hospitals were excluded. The ED’s surgical physician on call was questioned. If this physician was unavailable, an e-mail to the head of the ED was sent out. The questions were asked in German, French, or Italian, based on the respective language region in Switzerland.

Descriptive statistics were performed for counts and percentages.

Furthermore, a survey among specialists in oral and cranio-maxillofacial surgery was performed during the “36th Annual Meeting of the Swiss Society for Oral and Cranio-Maxillofacial Surgery” via online voting (StrawPoll [23]). All participants were asked if they knew about dental and maxillofacial algorithms in their EDs (Table 1).

## 3. Results

We received 81 answers from the 89 EDs approached, corresponding to a response rate of 91%. Eight answers were received by e-mail and 73 by telephone. Four answers were received from a level 1 hospital (university hospital), 48 from level 2 hospitals (center hospital), 13 from level 3 and 4 hospitals, and two answers were received from level 5 hospitals (small community hospitals).

The majority, with 62 (77%) of the 81 responding EDs, use “medStandards”. A total of 42% (*n* = 34) of them completely rely on “medStandards”, whereas 35% (*n* = 28) have access to “medStandards”, plus their own or other algorithms/protocols. Sixteen percent (*n* = 13) do not have access to “medStandards” but use in-house or other algorithms/protocols. Seven percent (*n* = 6) do not have electronic algorithms at all. The 7% without access to any algorithms/protocols stated that they are not interested in using algorithms.

**Figure 2:**
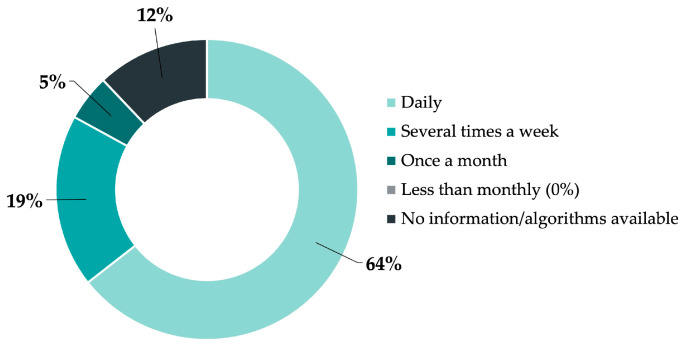
Frequency of use of electronic algorithms in Swiss emergency departments (EDs).

Figure 2 shows the use of algorithms in general; the majority, with 64%, use algorithms daily.

**Figure 3:**
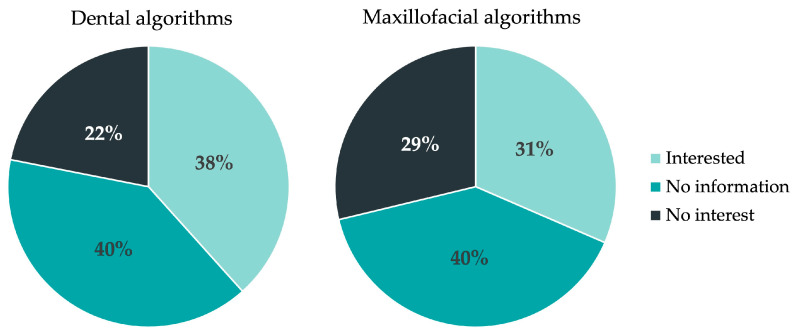
Demand for dental and maxillofacial algorithms in Swiss EDs.

For maxillofacial and dental algorithms, eight respondents (10%) stated they have maxillofacial respective dental algorithms (“medStandards”). Figure 3 shows the demand among the 73 respondents (90%) who answered that they have neither dental nor maxillofacial algorithms. Twenty-two percent had no interest in the usage of dental algorithms and 29% had no interest in maxillofacial algorithms.

**Figure 4:**
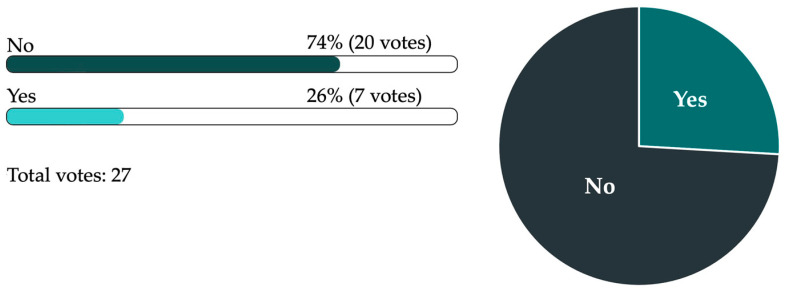
Results from the online survey among specialists in oral and maxillofacial surgery during the 36th Swiss Congress of Oral and Cranio-maxillofacial Surgery regarding their knowledge about the existence of dental and maxillofacial “medStandards”.

At the 36th Swiss Congress of Oral and Cranio-maxillofacial Surgery, we received 27 answers from the participants. Twenty-six percent knew there were dental and maxillofacial “medStandards” and 74% did not know about the availability of these algorithms (Figure 4).

## 4. Discussion

EDs, in general, suffer from a high workload. Algorithms can help to support the ED staff and have the potential to reduce decision-making time in acute situations.

In EDs, facial traumas are seen regularly. Overall, facial trauma is a common injury worldwide [1,2,3]. Causes leading to facial fractures differ geographically. In Switzerland, ground-level falls are the main causes of presentation to the ED with facial fractures [24,25]. In other regions, such as Turkey [26] and Latin America [27], traffic accidents are the most common causes. In North America [28], Australia [29], New Zealand [30], and South Africa [31], assaults are the major causes of facial fractures. While the final treatment of such fractures may vary between geographic regions, the basic work-up and primary treatment is essentially identical. The same observation might apply to odontogenic abscesses, another common cause for ED presentation.

Our study showed that if algorithms are available, they are mainly used on a daily basis. If not available, there is a particular demand for dental algorithms. This can be explained by the low knowledge of ED physicians concerning dental medicine [11] and the high incidence of dental trauma [9]. The dental trauma algorithm (Figure 1), as well as the other algorithms, explain in detail the workflow from initial assessment to immediate measures, correct diagnosis, specific treatment, and further procedures/follow-up. The algorithms contain main- and partial sub-slides, as well as a part containing detailed explanations. A section with further information on the respective topic is also available.

In detail, the information section of the dental trauma algorithm contains paragraphs about definitions, classification, dental terminology, tooth anatomy, dental scheme in adults and children, epidemiology, risk factors, complications, prognosis, prophylaxis, patient information, and information on consultation/referral to an oral and maxillofacial surgeon or dentist. It is based on the current literature in this field and according to the doctrine of the Faculty of Dentistry of the University of Basel. The information part in the dental trauma algorithm addresses physicians without dental knowledge in particular. Regarding dental trauma and dental emergency situations in general, it is important for an emergency physician to have in mind and recognize potentially life-threatening complications, such as a floor of the mouth hematoma; to be familiar with basic treatment modalities; and to be able to assess the urgency of referral to a dentist/oral and maxillofacial surgeon. Basic knowledge such as the correct storage of avulsed teeth in a tooth rescue box and modalities to control oral bleeding should be present.

Treatment of dental traumas in EDs can be a challenging task, particularly without special dental instruments, such as hooks, dental mirrors, a powerful light, compressed air, suction, etc. It is helpful to have access to a specially prepared dental trauma kit and a dental unit, especially for trauma splinting. For hospitals with an oral and maxillofacial surgeon/oral surgeon on call, this is a considerable option to enable them to be more efficient. At the least, the availability of a tooth rescue box—a small container with a special cell-culture medium [32,33]—at an ED is highly recommended. In Switzerland, many schools and public swimming pools do have tooth rescue boxes as standard first aid equipment and staff there should have knowledge on how to act in a tooth emergency situation [34]. The average patient usually has no or only rudimentary knowledge on how to transport a fractured or avulsed tooth until further treatment. It is vital that the tooth is stored in a tooth rescue box immediately upon arrival. Insufficient transport media would be saliva, mouthwash, or any other fluid other than isotonic saline solution/UHT (ultra-high temperature) milk, or dry storage [35]. Therefore, the recommendation would be to put the tooth in UHT milk or just save it from drying out by putting it in cling film since these are usually available in most households [35]. The survival rate of the periodontal ligament cells in a tooth rescue box is at least 24 h; comparable findings for cooled UHT milk storage could be found and cling film can also be used as an alternative for preservation up to six hours [35]. Avulsed teeth can be pretreated with tetracycline/dexamethasone as additives within the tooth rescue box to support the antiresorptive effect [33]. After the repositioning of an avulsed tooth, or if a tooth is only loosened but not completely avulsed, splinting is necessary to increase the survival rate of the tooth as well as the patient’s comfort [36]. Different options for splinting are available [36]. It would be desirable to use a semi-rigid splint such as a TTS (Titanium Trauma Splint Modus^®^, Medartis AG, Basel, Switzerland), but if this is not available, a simple either twisted or slightly thicker wire can also be used. For the fixation of a TTS or a wire, a bonding agent and a light curing composite are necessary. Most importantly, this fixation technique requires a dry tooth crown surface otherwise the fixation material will not work. This can be difficult to achieve in a traumatized, bloody oral cavity. Furthermore, it can be difficult to judge the correct position of teeth in this situation, which also requires experience and knowledge in prosthodontics, orthodontics, and oral surgery to restore the correct occlusion. Intruded teeth can be difficult to reposition and often need to be extruded with a forceps. Due to the complexity of these procedures, this should be performed by an experienced specialist.

Since the field of facial trauma is more complex, six algorithms are available for physicians to gain an overview of differential diagnoses and their red flags. Concomitant injuries, such as intracranial bleeding, need to be ruled out first, as do airway obstruction, cervical spine fractures and retrobulbar hematoma. The algorithms of blunt midface trauma and differential diagnosis in facial trauma provide help to find the correct main algorithm. The algorithm about LeFort fractures and zygomatic/orbital fracture indicates the importance of examining the patient for a potential vision-threatening retrobulbar hematoma and to involve the ophthalmologists to check the affected eye in case of orbital involvement. The algorithm about mandibular fractures divides the main diagnoses into fractures involving the tooth-bearing region of the mandible and the ones outside the dentition and points out the importance of examining the occlusion. A second fracture in a related region needs to be excluded. This can be a more common fracture, such as condyle fractures, if a mandible body fracture is present but also trauma in neighboring regions such as the external auditory canal. Furthermore, airway obstruction needs to be considered. The nasal bone fracture algorithm focuses especially on hematoma of the nasal septum, as a specific red flag.

Exemplarily, the odontogenic abscess algorithm supports the correct classification of different abscesses and highlights red flags. There is a variety of odontogenic abscesses. Small abscesses such as submucosal abscesses, periodontal abscesses, or other local abscesses can mostly be treated quickly by incision under local anesthesia. Depending on the size of the abscess and local practice preferences, a silicon drain, latex-/powder-free gloves (off-label use), or meshes with antiseptic or antibiotic coating can be used for the drainage of an abscess and to rinse for further treatment. At the University Hospital Basel, a small and soft silicone tube is usually inserted and fixated with stiches to ensure the drainage. On localized abscesses, the use of systematic antibiotics is controversial and therefore is only mentioned generally in the “medStandards” algorithm. Larger abscesses affecting fascial spaces may need to be incised and drained from extraoral under general anesthesia. For these patients, additional intravenous antibiotic treatment and a hospitalization is recommended. Incisions from extraoral carry the risk of damaging the facial nerve. The lingual and mental nerves may also be located in the surgical area and could be harmed; therefore, if possible, the patient should be consenting.

An administrative algorithm for dental and maxillofacial imaging is internally available. The decision to add an algorithm for dental and maxillofacial imaging was made since the consistency in dental imaging differed enormously. The diagnostic X-ray should be most informative. On the other hand, radiation dose reduction or protection, as emphasized in the ALARA principle (“As Low As Reasonably Achievable”) [37], must be respected.

A further algorithm should be created dealing with oral bleeding. Due to the increasing use of anticoagulants [38], there is a steady increase in patients dealing with minor (post-operative) bleedings such as constant blood loss out of the periodontal gap after a professional teeth cleaning or minor cuts after eating hard food. These examples might be manageable without major interventions. A swab dripped in tranexamic acid and a constant pressure for a longer time might be sufficient. Silver nitrate sticks can also be used for their astringent effects to stop minor bleeding in superficial mucosal lesions. Bleedings after tooth extractions or tumor bleeding can lead to life-threatening blood loss. Depending on the type of bleeding as well as the type of anticoagulation used, different treatment options are available. Diagnostically, blood values, especially coagulation status, must be checked, and blood pressure has to be controlled. For some anticoagulants, there are specific antidotes available. Depending on the type of the anticoagulant, the substitution of antagonists such as vitamin K for phenprocoumon overdose or andexanet alfa (Ondexxya^®^) as an antidote for rivaroxaban/apixaban can be administered. Among other factors, liver and renal function as well as dietary factors can influence blood coagulation and should be evaluated. Locally, hemostatic agents (tranexamic acid, absorbable hemostatic gelatin sponge (e.g., Spongostan^TM^), absorbable hemostatic cellulose (e.g., Tabotamp^®^), and epinephrine containing local anesthesia) can be used but might not be sufficient in certain situations; therefore, the administration of prothrombin complex concentrate (PCC) or fresh frozen plasma (FFP), for example, can be considered.

While previous reported response rates among physicians are critically low [39,40], our study had an excellent response rate of 91% compared to other telephone surveys [41,42]. This could be due to the short questionnaire or the direct approach from physician to physician. In general, written and telephone surveys have a higher response rate than web-based surveys [40,43]. Published data about response rates vary between 13% for online surveys [44] and 84% for postal surveys [41]. For surveys focusing on emergency physicians, the reported response rates range from 13% to 57% [44,45,46,47,48].

As a limitation, there is a non-response bias in our study resulting from the fact that only hospitals in Italian- and French-speaking regions did not reply. Various studies have shown differences between the different language areas within Switzerland concerning, for example, attitude [49,50] and preventive behavior [51]. As “medStandards” is only available in German and English, it can be expected that the rate of availability and use of “medStandards” would be lower with more respondents from the French- and Italian-speaking parts of Switzerland.

A response bias could originate from the single interview conducted for each hospital. However, the senior physician on call can be expected to know about the availability of algorithms/protocols.

While some algorithms, such as “medStandards”, focus on emergency medicine in a broader context, others focus on internal medicine [18]. Algorithms, such as the ones from the “Emergency Medicine Kenya Foundation”, provide free ED protocols available online, including trauma algorithms [20]. The newest addition to ED protocols are possibly the “Oxford University Press” algorithms, including a chapter about maxillofacial trauma [19]. Specialized guidelines focusing on facial fracture/trauma can be found online from the “AO Foundation” (Association of the study of the Study of Internal Fixation) [52]. Guidelines for trauma and emergency situations are available in German from the “AWMF” (Association of the Scientific Medical Societies in Germany) [15]. This guideline collection also offers specific guidelines for oral and maxillofacial surgery, including dental trauma, temporomandibular joint luxation, as well as diagnostics and therapy for midface fractures. Unfortunately, most of these guidelines are in text form and not in an algorithmic design, which is a disadvantage in acute care. In Switzerland, there are other general clinical algorithms available, for example, from a community hospital [53] and a university hospital [54]. However, they do not cover all everyday situations, particularly no dental or maxillofacial topics, and are not available in English. Some algorithms in the field of dental and maxillofacial surgery in English were identified in a literature review [55,56,57,58,59,60]. Even more clinical guidelines are available in text form [61,62,63,64,65,66,67].

Even if guidelines are available, they might not be appropriately used. In this study, a certain disparity was evident: while 77% of the respondents reported having “medStandards” at their disposal (which would include maxillofacial and dental algorithms), only 10% reported having access to maxillofacial and dental algorithms. This means that 67% of the participants did not know the contents of the algorithms available to them. This lack of awareness should be addressed.

It is of major interest that algorithms are always up to date and applicable. Direct feedback from the practitioner can provide relevant suggestions for improvement. There is an obvious need for application research in this field and in the usability and feasibility of algorithms/protocols, which should be assessed in more detail. As more of these applications are spreading, the content and the base of evidence is another concern. Since textbooks may be outdated when published, and guidelines in text form are rarely read in their entire length, it can be prognosticated that algorithms are on the rise, particularly in emergency medicine, as the need for rapid and reliable information is obvious.

## 5. Conclusions

In conclusion, the straightforward availability of dental and maxillofacial emergency algorithms is beneficial in everyday clinical practice, especially for emergency physicians who wish to gain a quick overview on a dental and maxillofacial condition and to support decision making at triage, during diagnostic work-up, and for initial therapy. Unfortunately, information about the existence of such algorithms does not seem to reach every practitioner.

## Figures and Tables

**Table 1:** Telephone survey among doctors in Swiss emergency departments (ED).

Questions and Corresponding Answer Possibilities	
Are electronic algorithms/protocols, such as “medStandards”, available in your ED?	
No Yes	Is there any demand for algorithms?
Do you have algorithms/protocols regarding maxillofacial surgery in your clinic?	
Yes No	Own protocols ready for distribution? Is there a demand for maxillofacial algorithms?
Do you have dental algorithms/protocols in your clinic?	
Yes No	Could you send them to us? Would there be a demand for such algorithms?
Do you have own algorithms/protocols, or a commercially available product?	
Own/in-house algorithms/protocols Commercially available algorithms/protocols	Which brand?
How often do you use such algorithms/protocols in general?	
Daily Several times a week Monthly Less than monthly	

## Data Availability

The raw data presented in this study are available on request from the corresponding author.
